# Supplementary material for: Metal and Ligand Effect on the Structural Diversity of Divalent Coordination Polymers with Mixed Ligands: Evaluation for Photodegradation
Source: Molecules. 2023 Feb 27;28(5):2226. doi: 10.3390/molecules28052226 (PMC10004691; doi:10.3390/molecules28052226)
Supplement: Supplementary file 1 [file molecules-28-02226-s001.zip › molecules-2230689-Supplementary Materials.pdf]

Supplementary Materials

Metal and Ligand Effect on the Structural Diversity of Divalent Coordination Polymers with Mixed Ligands: Evaluation for Photodegradation

Manivannan Govindaraj [1], Shih-Ying Zhong [1], Chia-Her Lin [2,*] and Jhy-Der Chen [1,*]

[1] Department of Chemistry, Chung-Yuan Christian University, Chung Li, Taoyuan City 320, Taiwan; manivannanjent@gmail.com (M.G.); 4ying4ying@gmail.com (S-Y.Z.)
[2] Department of Chemistry, National Taiwan Normal University, Taipei 106, Taiwan
* Correspondence: chiaher@ntnu.edu.tw (C.-H.L.); jdchen@cycu.edu.tw (J.-D.C.); Tel.: +886-3-265-3351 (J.-D. C.)

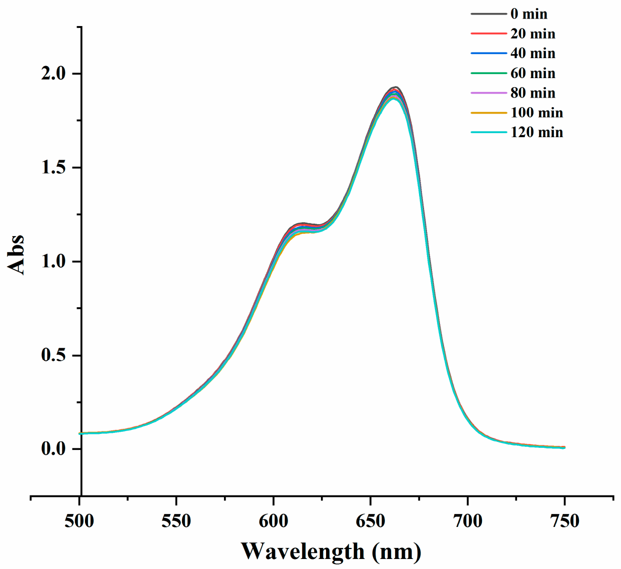

Figure S1. UV-vis spectra of the tube 1.

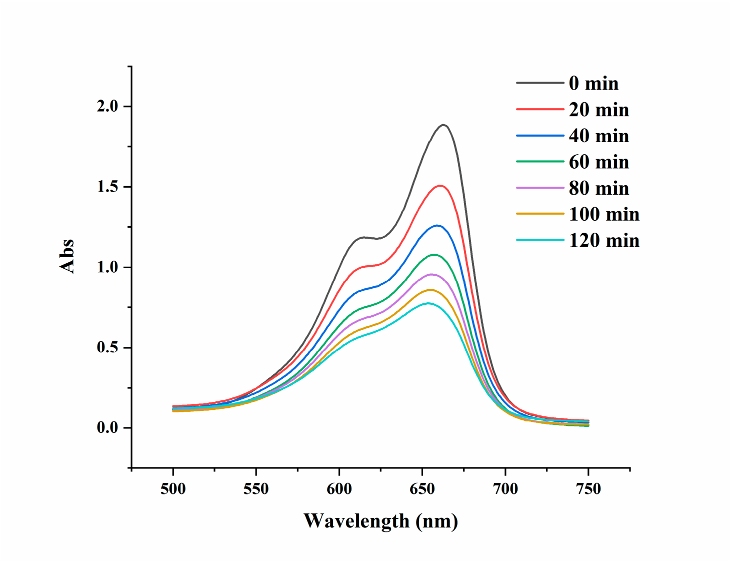

Figure S2. UV-vis spectra of the tube 2.

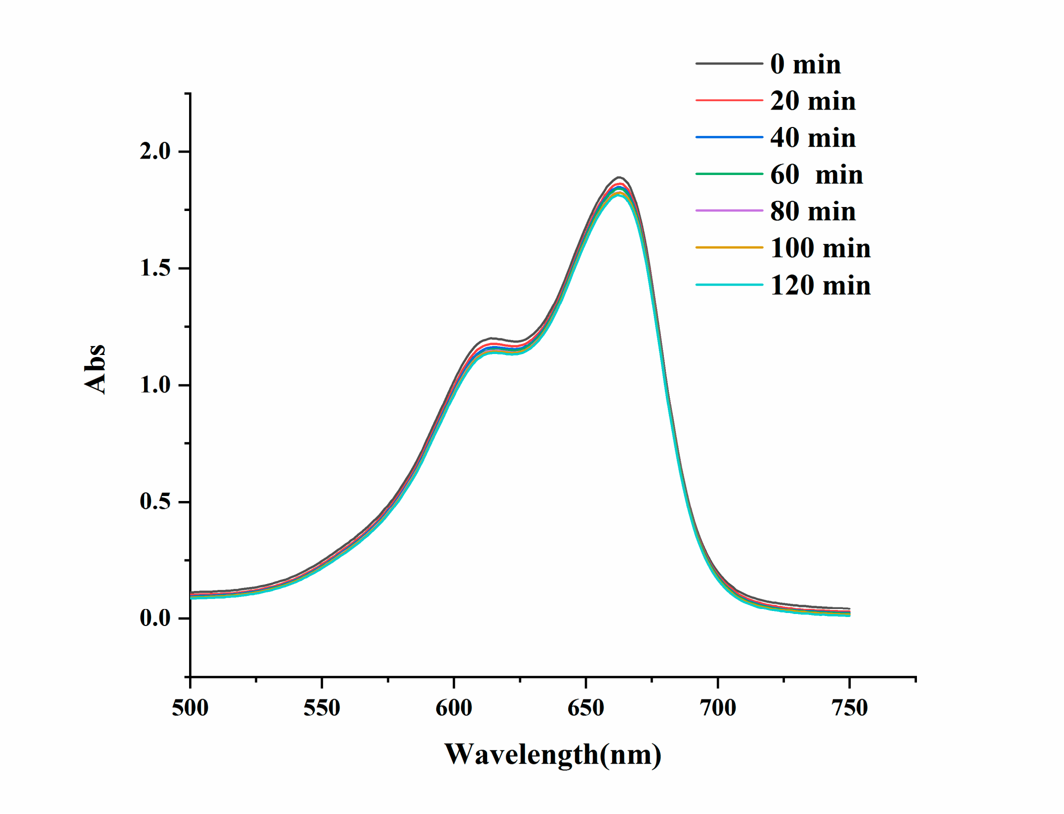

Figure S3. UV-vis spectra of tube 3 for complex **1**.

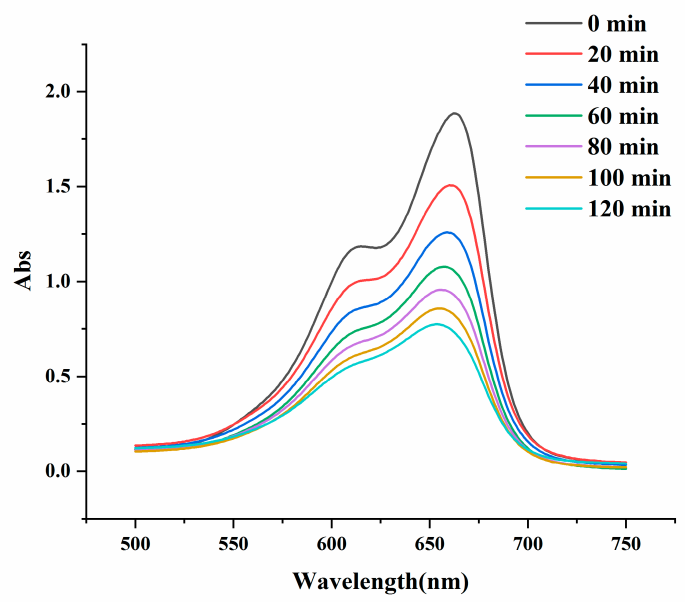

Figure S4. UV-vis spectra of tube 4 for complex **1**.

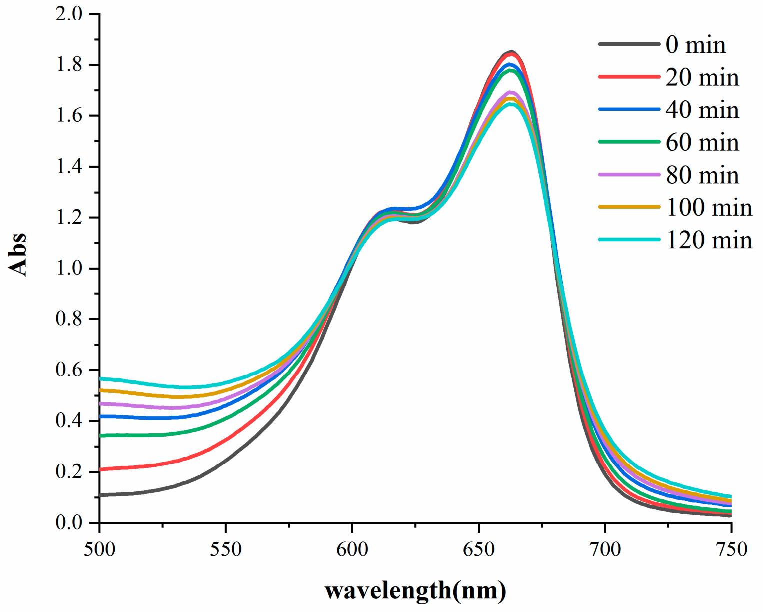

Figure S5. The UV-vis spectra of tube 3 for complex **2**.

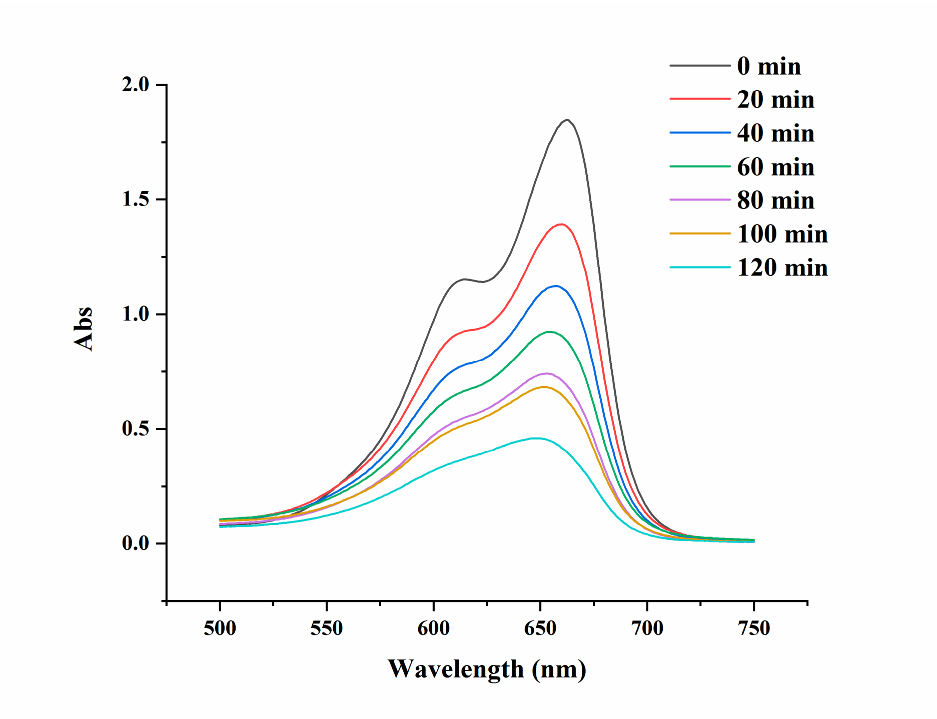

Figure S6. UV-vis spectra of tube 4 for complex **2**.

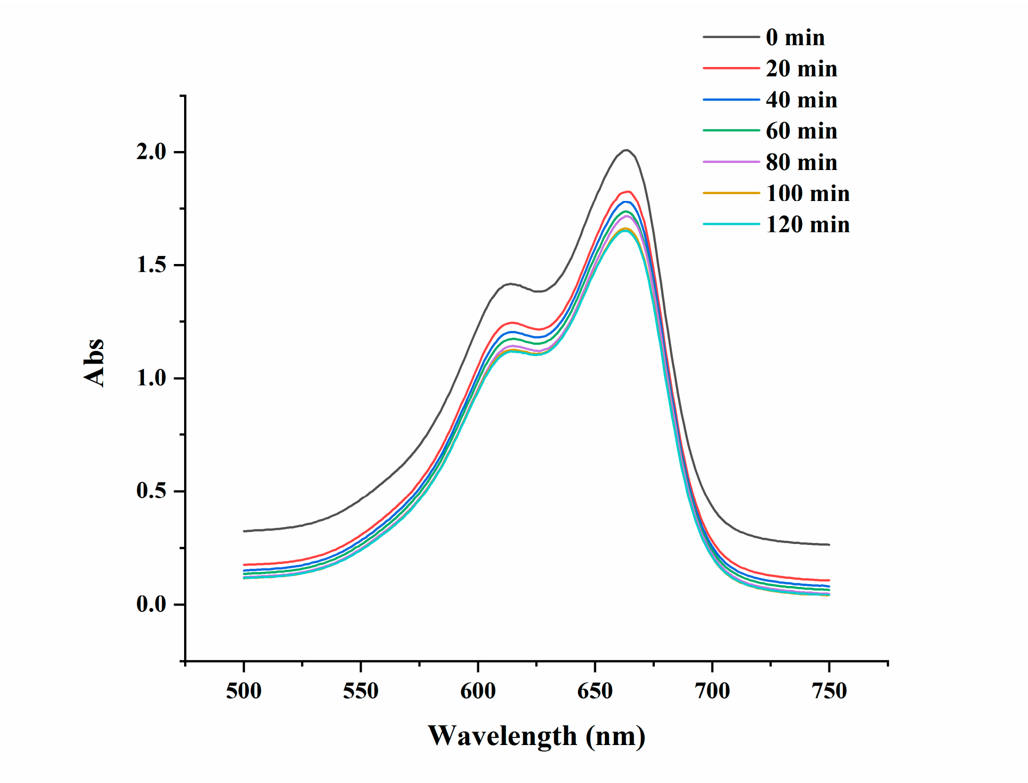

Figure S7. UV-vis spectra of tube 3 for complex **3**.

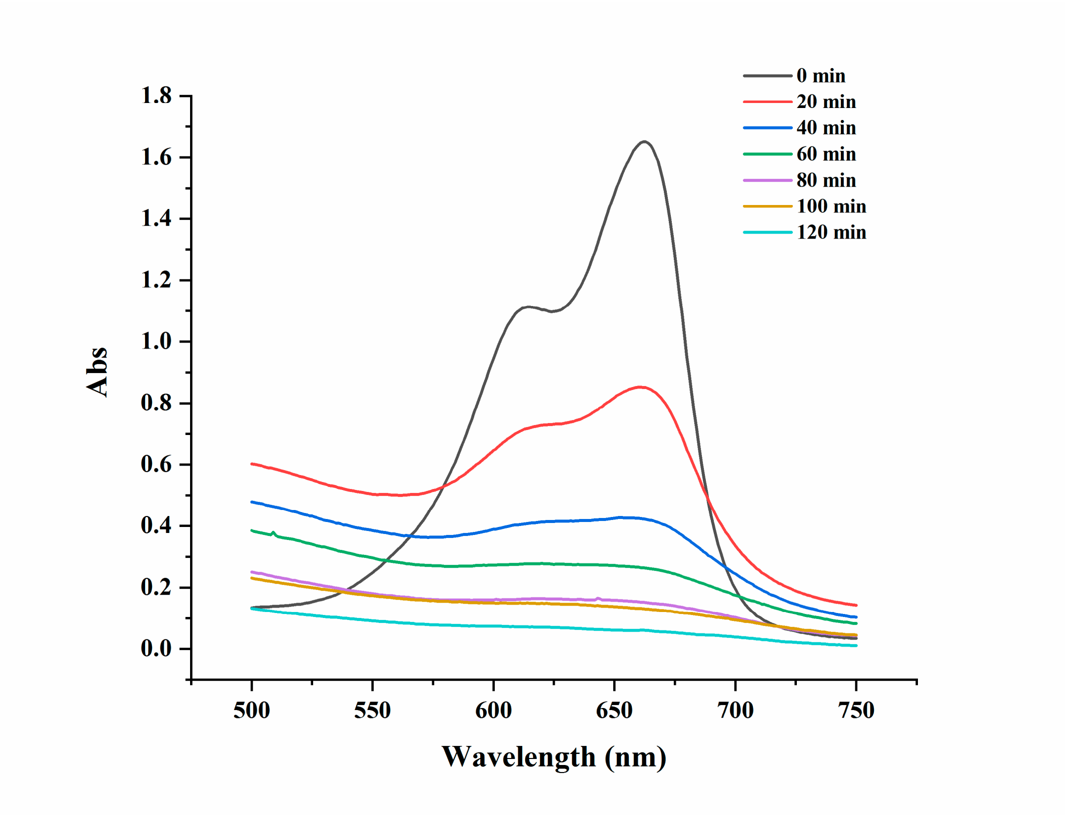

Figure S8. UV-vis spectra of tube 3 for complex **3**.

Table S1. Degradation efficiency (DE) with the mean values and standard deviations for MB solution under catalytic experiment.

**Time interval**	**1st**	**2nd**	**3rd**	**Mean**	**Standard deviation**
0	100.00	100.00	100.00	100.00	0
20	99.09	99.17	99.32	99.20	0.12
40	98.88	98.76	98.91	98.85	0.08
60	98.13	98.08	98.33	98.18	0.13
80	97.44	97.56	97.71	97.57	0.13
100	96.91	96.94	97.55	97.13	0.36
120	96.86	96.58	97.45	96.96	0.44
DE	3.14	3.42	2.55	3.04	

Table S2. Degradation efficiency (DE) with the mean values and standard deviations for MB solution + H_2O_2 under catalytic experiment.

**Time interval**	**1st**	**2nd**	**3rd**	**Mean**	**Standard deviation**
0	100.00	100.00	100.00	100.00	0
20	82.80	83.94	87.24	84.66	2.31
40	70.34	73.39	76.44	73.39	3.05
60	60.91	64.79	67.30	64.34	3.22
80	53.66	59.00	63.03	58.56	4.70
100	47.14	52.81	56.20	52.05	4.58
120	41.49	47.19	50.92	46.53	4.75
DE	58.51	52.81	49.08	53.47	

Table S3. Degradation efficiency (DE) with the mean values and standard deviations for complex **1** under catalytic experiment.

**Time interval**	**1st**	**2nd**	**3rd**	**Mean**	**Standard deviation**
0	100.00	100.00	100.00	100.00	0.00
20	97.65	98.09	97.03	97.59	0.53
40	96.08	96.96	95.85	96.30	0.58
60	95.20	96.91	95.65	95.92	0.89
80	94.36	95.87	94.26	94.83	0.90
100	93.63	95.82	94.21	94.55	1.14
120	93.11	95.36	93.70	94.05	1.17

DE	6.89	4.64	6.30	5.95	

Table S4. Degradation efficiency (DE) with the mean values and standard deviations for MB solution + complex **1** + H_2O_2 under catalytic experiment.

**Time interval**	**1st**	**2nd**	**3rd**	**Mean**	**Standard deviation**
0	100.00	100.00	100.00	100.00	0
20	79.21	79.64	80.64	79.83	0.73
40	67.19	65.85	66.11	66.38	0.70
60	57.63	55.73	57.78	57.05	1.14
80	49.74	48.94	49.97	49.55	0.54
100	44.10	43.37	44.40	43.96	0.52
120	38.35	38.60	38.99	38.65	0.32
**DE**	61.65	61.40	61.01	61.35	

Table S5. Degradation efficiency (DE) with the mean values and standard deviations for MB solution + complex **2** under catalytic experiment.

**Time interval**	**1st**	**2nd**	**3rd**	**Mean**	**Standard deviation**
0	100.00	100.00	100.00	100.00	0.00
20	97.72	98.14	99.72	98.52	1.05
40	95.49	97.34	96.10	96.31	0.94
60	94.27	96.59	95.14	95.33	1.17
80	89.71	96.54	92.71	92.99	3.42
100	88.49	95.84	88.64	90.99	4.20

120	87.27	93.61	87.85	89.58	3.50
DE	12.73	6.39	12.15	10.42	

Table S6. Degradation efficiency (DE) with the mean values and standard deviations for MB solution + complex **2** + H_2O_2 under catalytic experiment.

**Time interval**	**1st**	**2nd**	**3rd**	**Mean**	**Standard deviation**
0	100.00	100.00	100.00	100.00	0.00
20	72.72	74.59	75.15	74.15	1.27
40	56.63	59.05	61.34	59.01	2.36
60	45.05	47.51	50.46	47.67	2.71
80	35.33	37.00	41.31	37.88	3.09
100	33.64	27.68	29.07	30.13	3.12
120	20.27	21.34	25.61	22.41	2.82
DE	79.73	78.66	74.39	77.59	

Table S7. Degradation efficiency (DE) with the mean values and standard deviations for MB solution + complex **3** under catalytic experiment.

**Time interval**	**1st**	**2nd**	**3rd**	**Mean**	**Standard Deviation**
0	100.00	100.00	100.00	100.00	0.00
20	99.50	90.84	90.04	93.46	5.24
40	89.20	88.60	87.68	88.49	0.76
60	87.94	86.46	86.31	86.90	0.90
80	86.23	84.47	85.97	85.56	0.95

100	85.08	82.73	84.54	84.12	1.23
120	84.17	82.18	84.35	83.57	1.20
DE	15.83	17.82	15.65	16.43	

Table S8. Degradation efficiency (DE) with the mean values and standard deviations for MB solution + complex **3** + H_2O_2 under catalytic experiment.

**Time interval**	**1st**	**2nd**	**3rd**	**Mean**	**Standard Deviation**
0	100.00	100.00	100.00	100.00	0.00
20	55.94	51.61	52.26	53.27	2.34
40	30.78	25.56	26.95	27.77	2.70
60	18.18	15.99	15.83	16.67	1.31
80	11.93	9.21	10.30	10.48	1.37
100	9.91	7.87	6.36	8.05	1.78
120	6.24	3.69	4.90	4.94	1.27
DE	93.76	96.31	95.10	95.06	

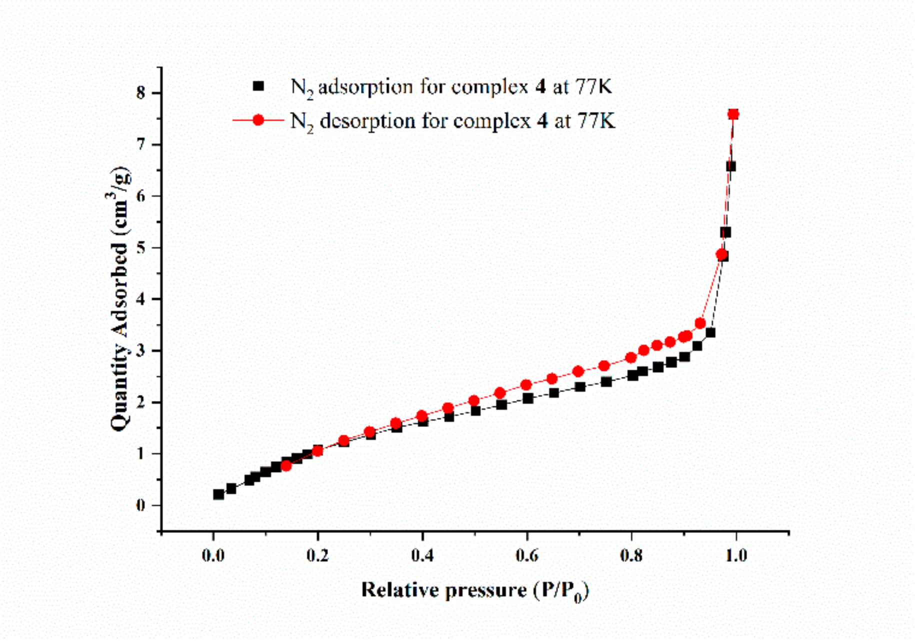

Figure S9. N_2 sorption isotherm for complex **1**.

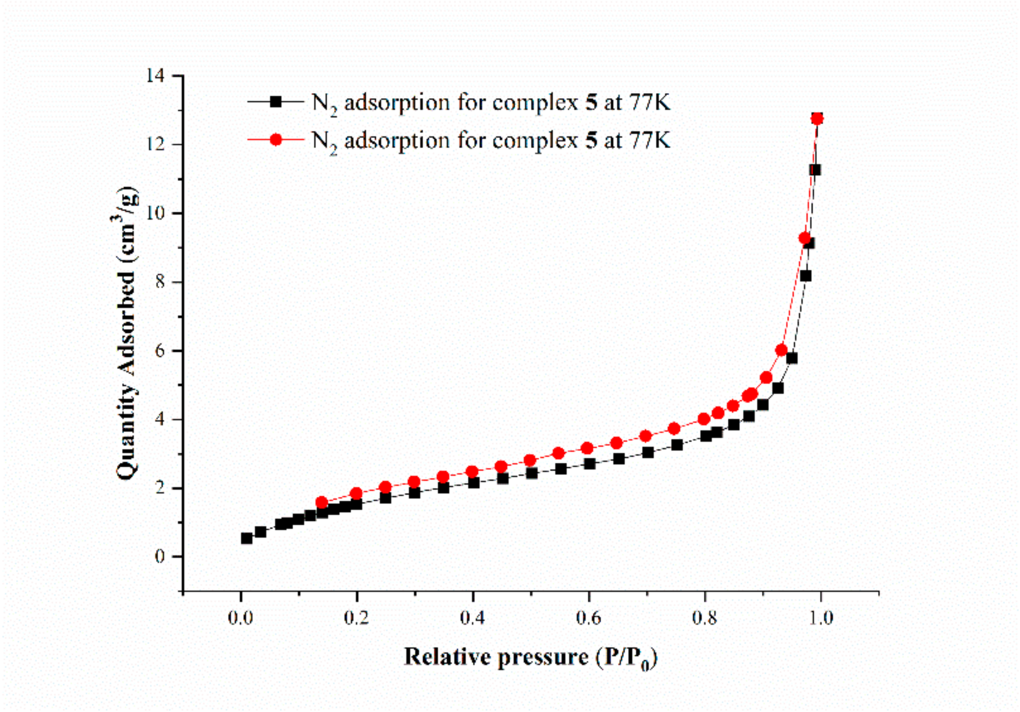

Figure S10. N_2 sorption isotherm for complex **2**.

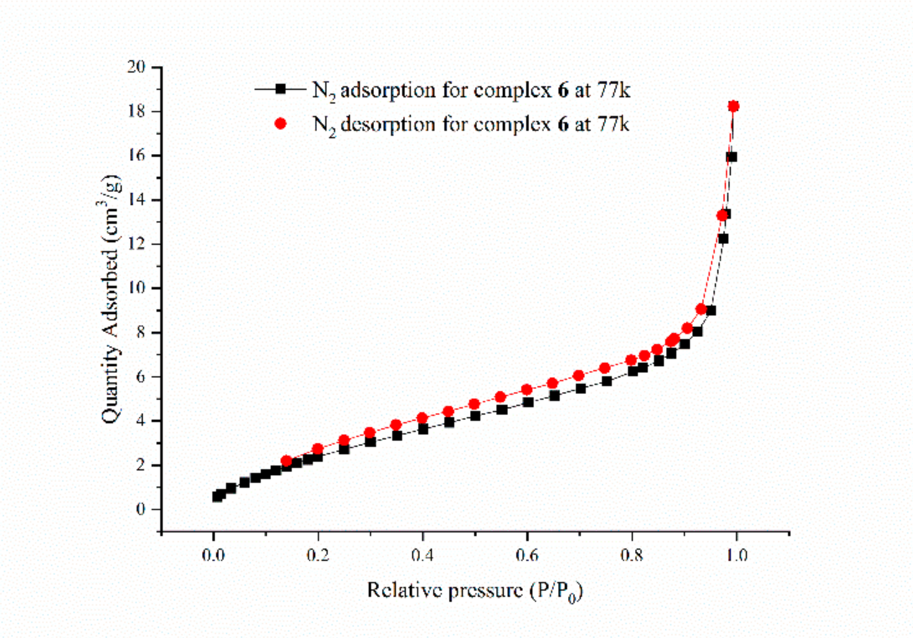

Figure S11. N_2 sorption isotherm for complex **3**.

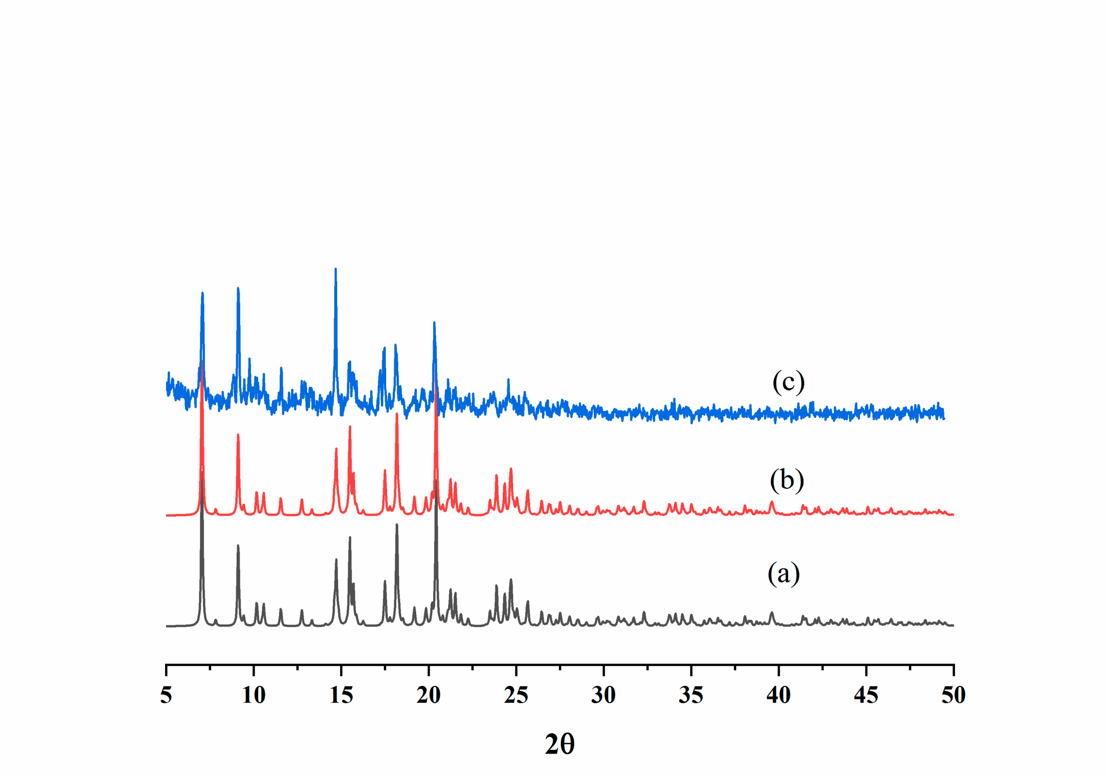

Figure S12. The PXRD pattern of **1** (a) simulated from single-crystal X-ray data, (b) as synthesized and (c) after photodegradation experiment.

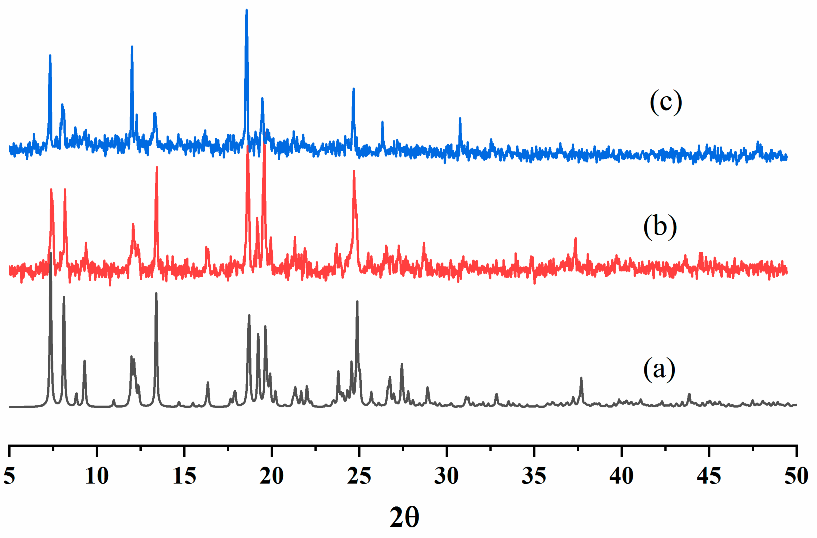

Figure S13. The PXRD pattern of **2** (a) simulated from single-crystal X-ray data, (b) as synthesized and (c) after photodegradation experiment.

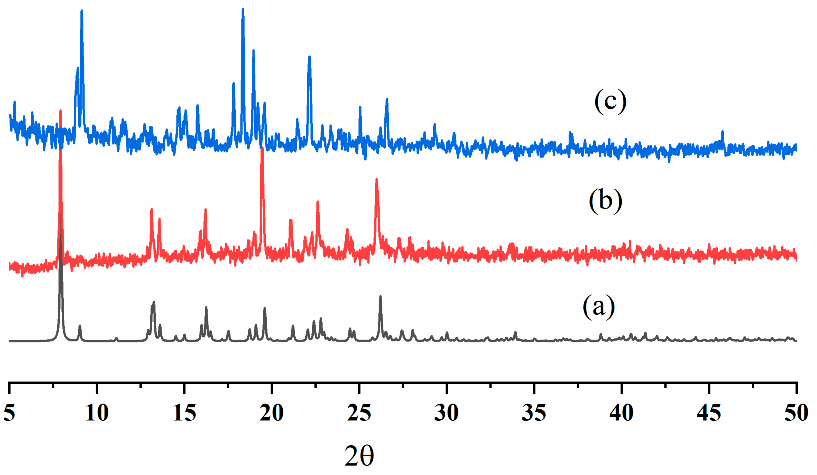

Figure S14. The PXRD pattern of **3** (a) simulated from single-crystal X-ray data, (b) as synthesized and (c) after photodegradation experiment.

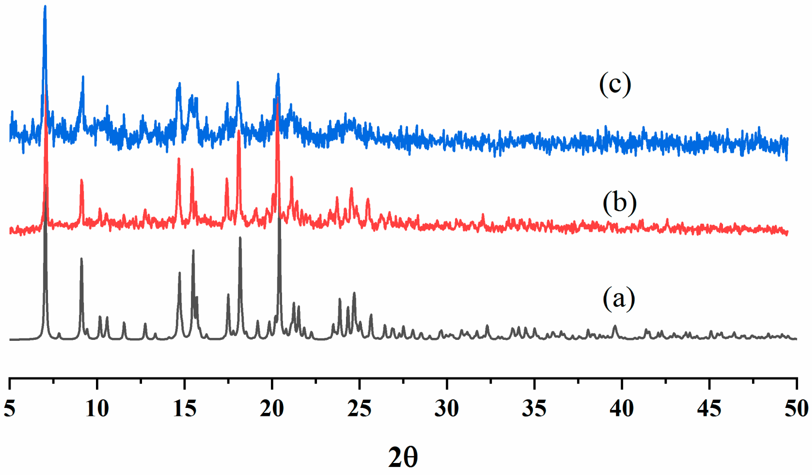

Figure S15. The PXRD pattern of **1**(a) simulated from single-crystal X-ray data, (b) as synthesized and (c) after nitrogen adoption experiment.

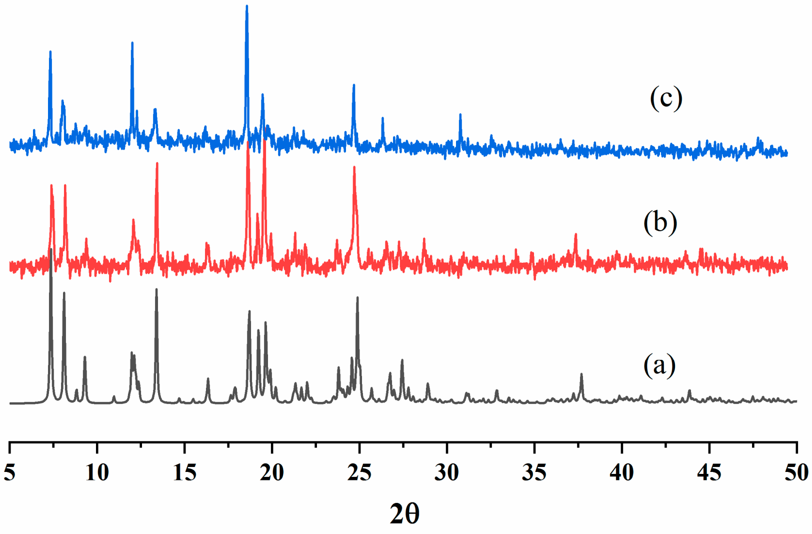

Figure S16. The PXRD pattern of **2** (a) simulated from single-crystal X-

ray data, (b) as synthesized and (c) after nitrogen adoption experiment.

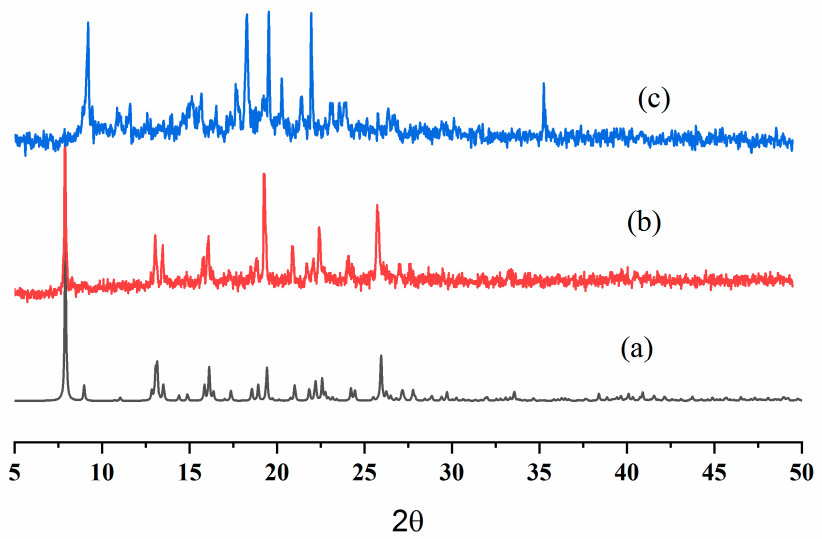

Figure S17. The PXRD pattern of **3** (a) simulated from single-crystal X-ray data, (b) as synthesized and (c) after nitrogen adoption experiment.

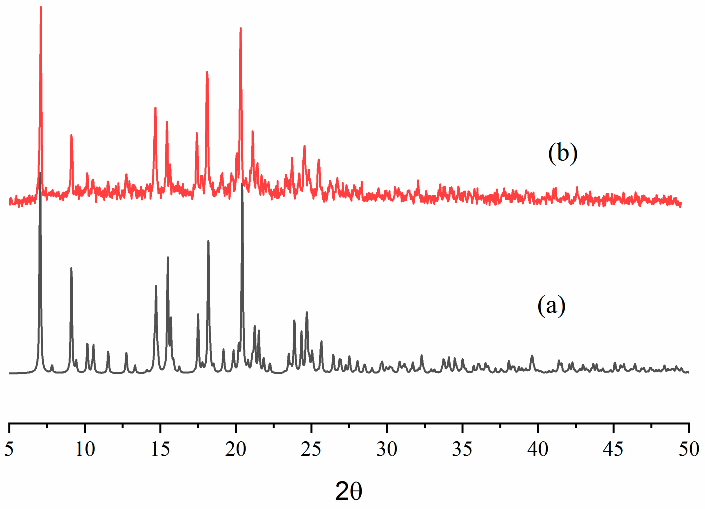

Figure S18. The PXRD pattern of (a) simulated from single-crystal x-ray data and (b) bulk materials as synthesized for complex **1**.

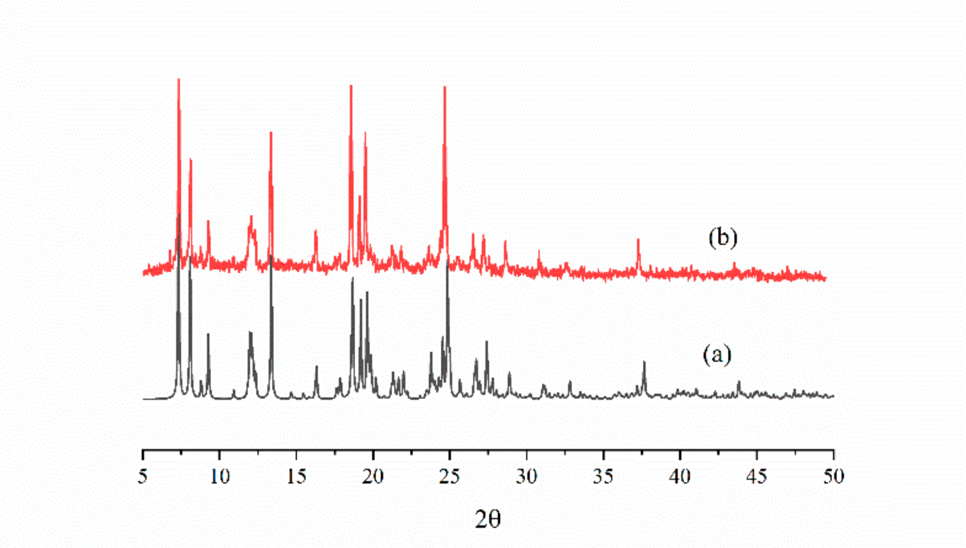

Figure S19. The PXRD pattern of (a) simulated from single-crystal x-ray data and (b) bulk materials as synthesized for complex **2**.

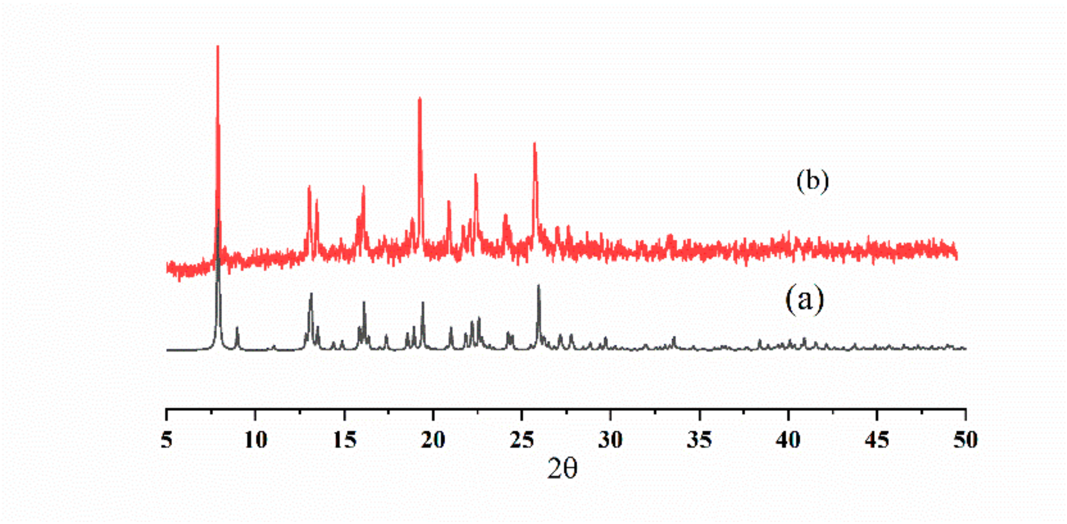

Figure S20. The PXRD pattern of (a) simulated from single-crystal x-ray data and (b) bulk materials as synthesized for complex **3.**

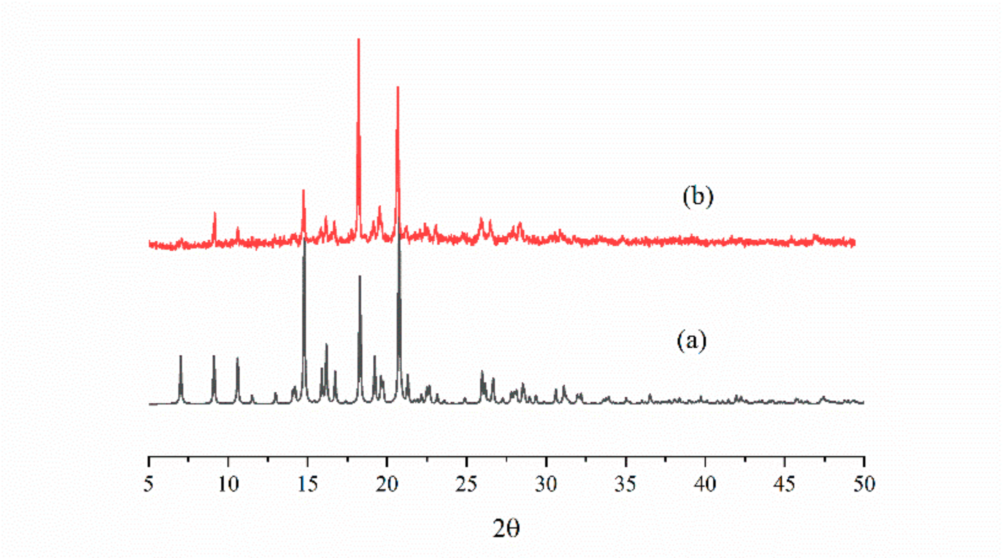

Figure S21. The PXRD pattern of (a) simulated from single-crystal x-ray data and (b) bulk materials as synthesized for complex **4**.

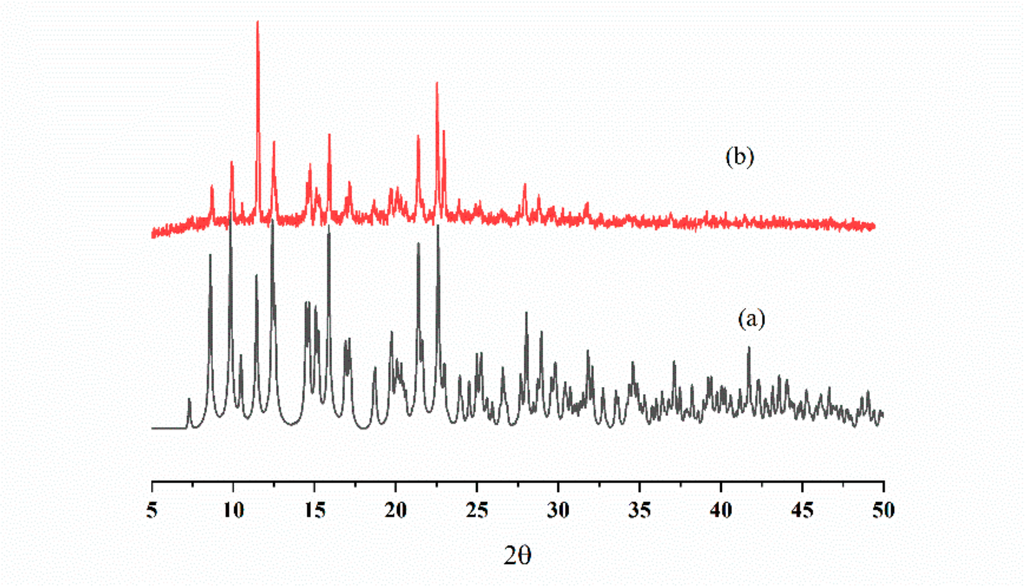

Figure S22. The PXRD pattern of (a) simulated from single-crystal x-ray data and (b) bulk materials as synthesized for complex **5**.

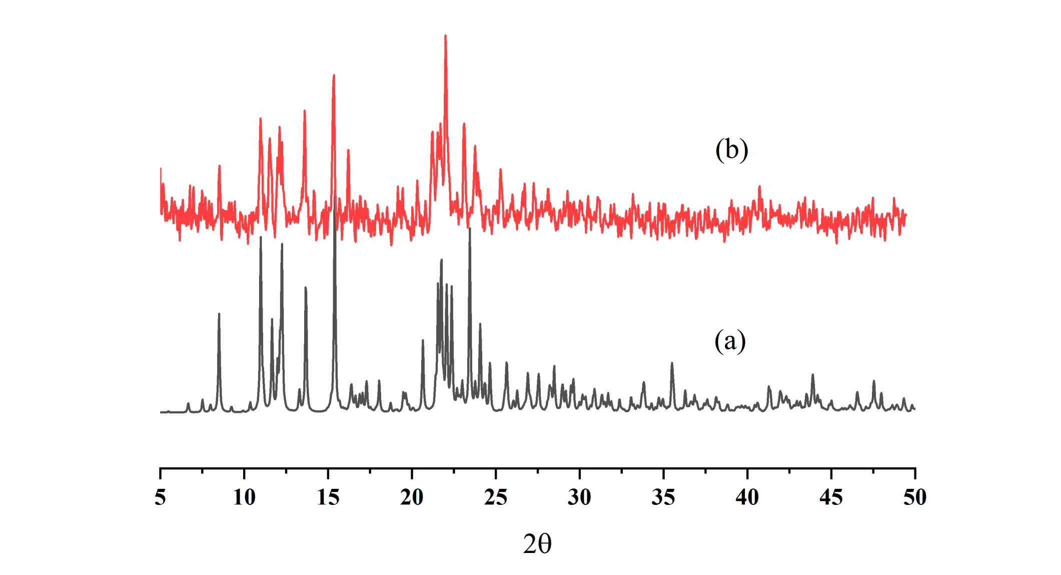

Figure S23. The PXRD pattern of (a) simulated from single-crystal x-ray data and (b) bulk materials as synthesized for complex **6**.

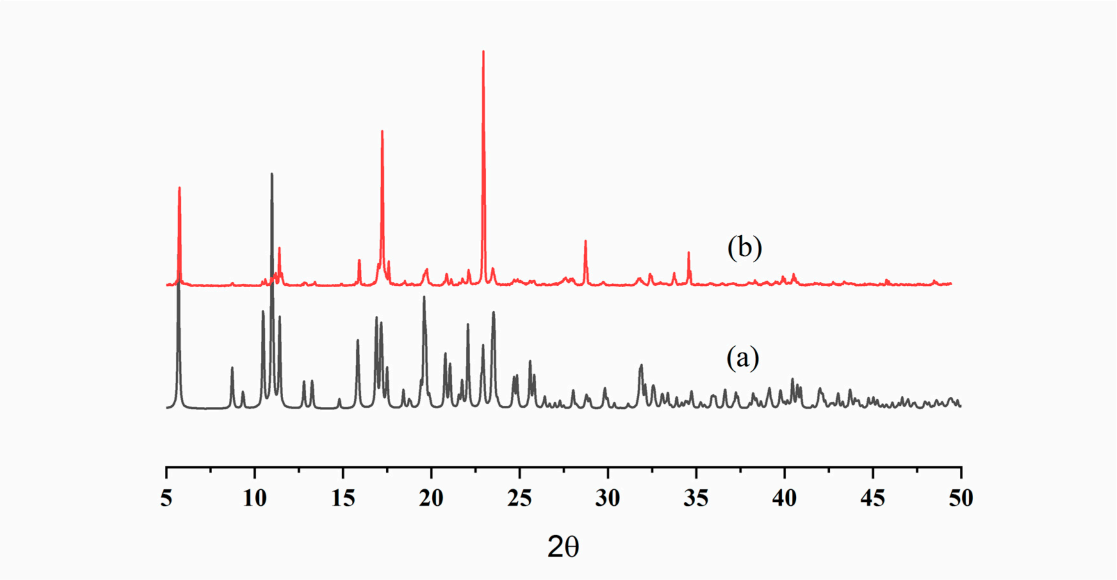

Figure S24. The PXRD pattern of (a) simulated from single-crystal x-ray data and (b) bulk materials as synthesized for complex **7**.

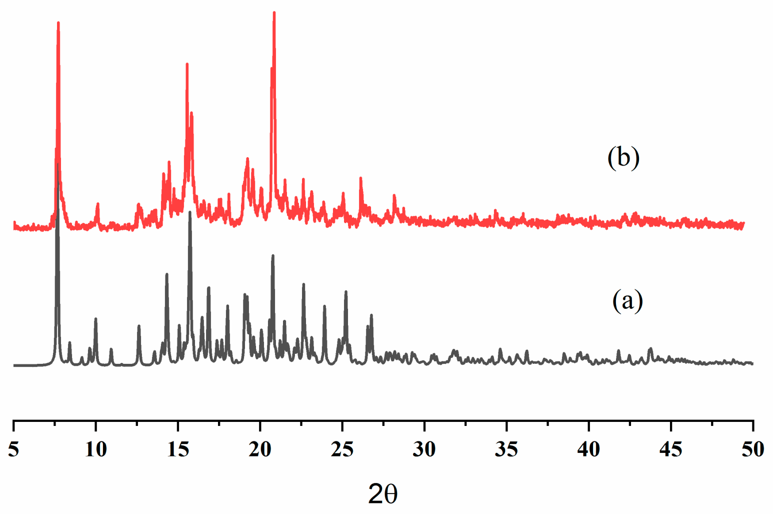

Figure S25. The PXRD pattern of (a) simulated from single-crystal x-ray data and (b) bulk materials as synthesized for complex **8**.

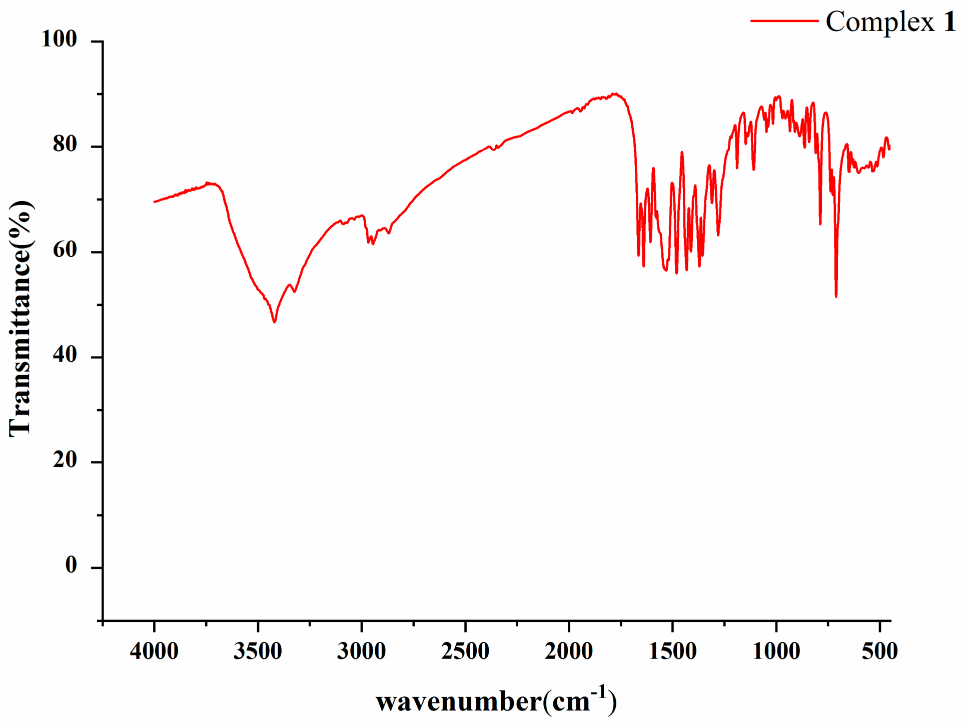

(a)

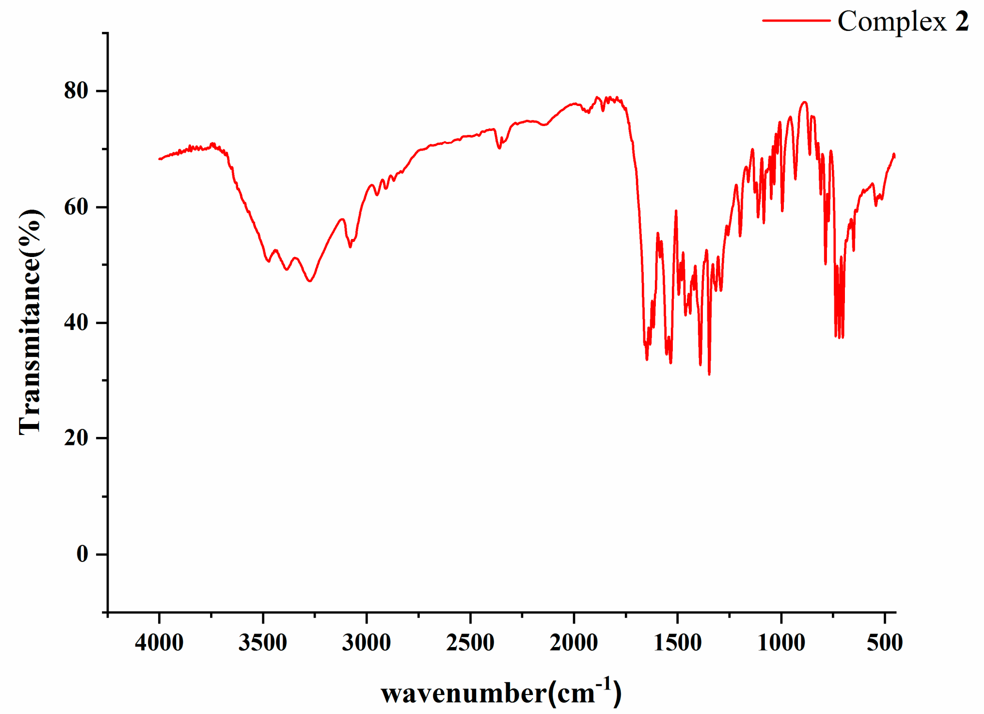
Complex 2
Transmitance(%)
80
60
40
20
0
4000
3500
3000
2500
2000
1500
1000
500
wavenumber(cm-1)

(b)

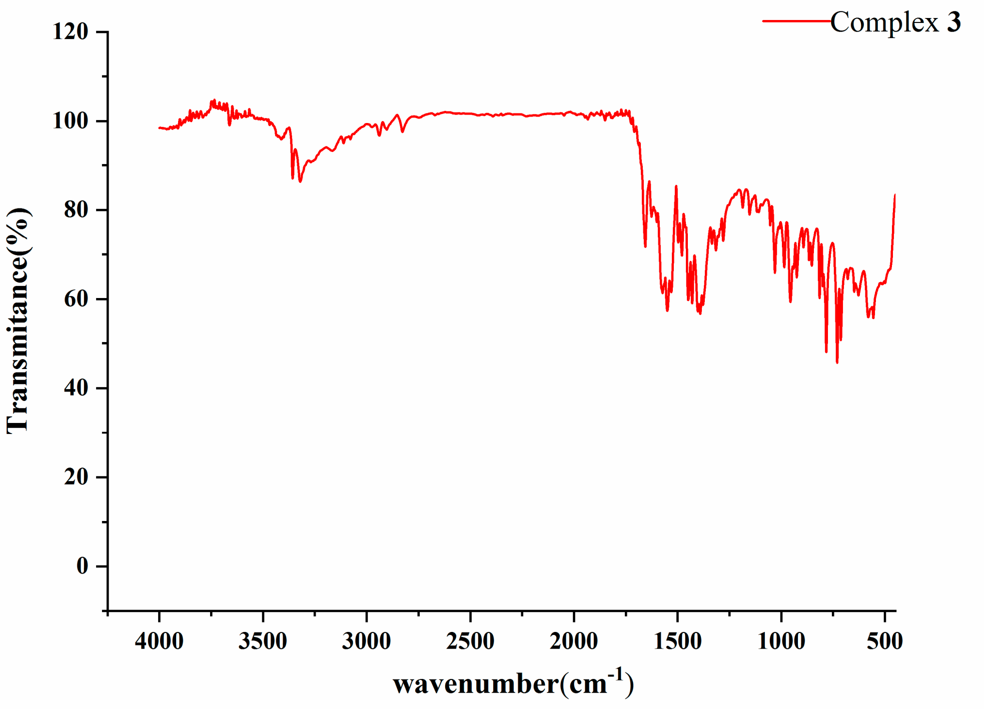
Complex 3
Transmitance(%)
120
100
80
60
40
20
0
4000
3500
3000
2500
2000
1500
1000
500
wavenumber(cm-1)

(c)

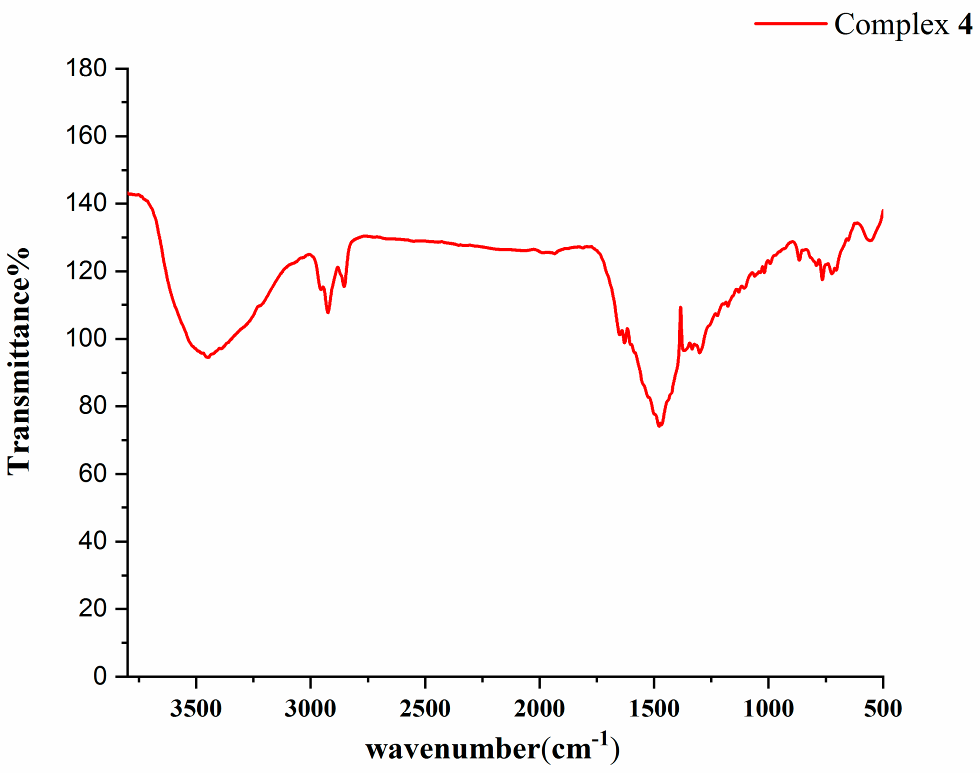
Complex 4
180
160
140
120
100
80
60
40
20
0
Transmittance%
3500
3000
2500
2000
1500
1000
500
wavenumber(cm^{-1})

(d)

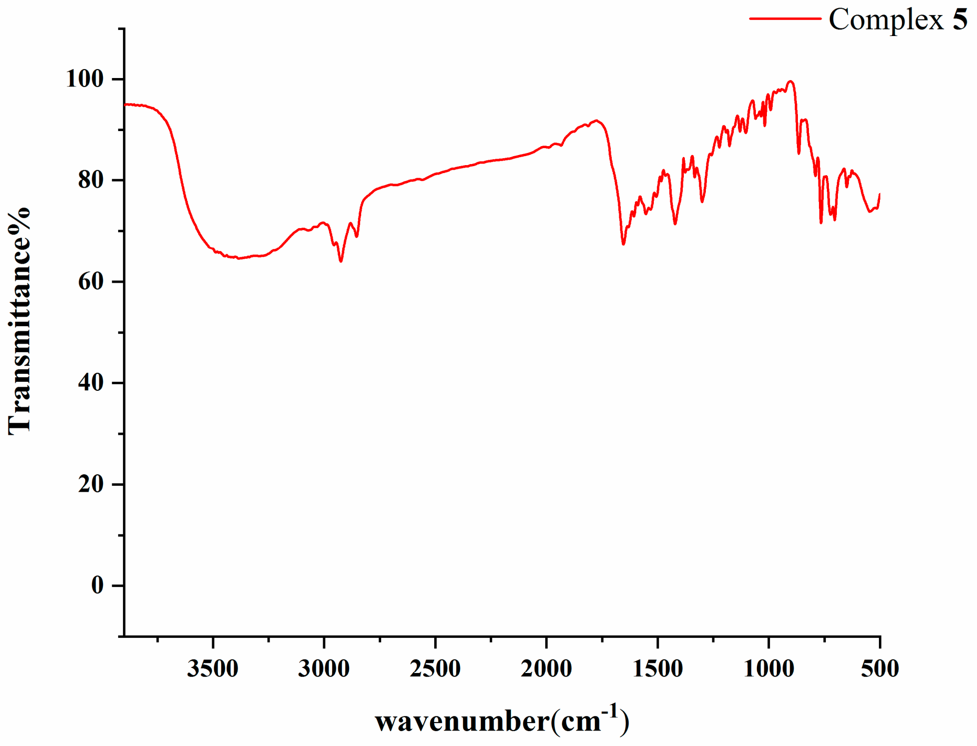
Complex 5
100
80
60
40
20
0
Transmittance%
3500
3000
2500
2000
1500
1000
500
wavenumber(cm^{-1})

(e)

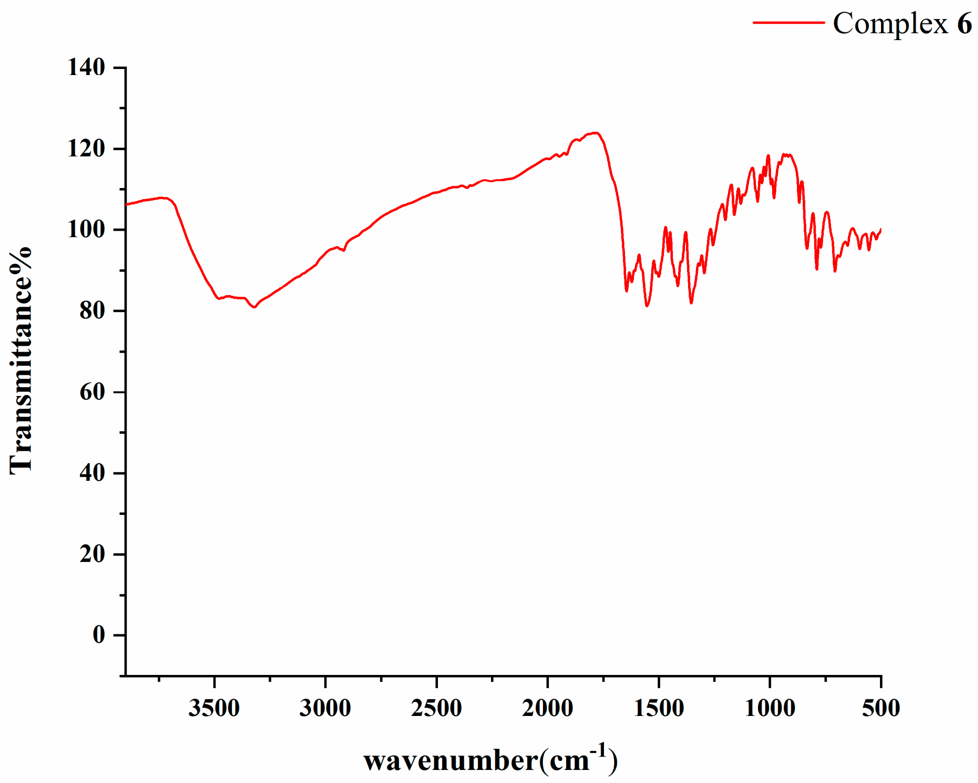

Complex 6
Transmittance%
140
120
100
80
60
40
20
0
3500
3000
2500
2000
1500
1000
500
wavenumber(cm-1)

(f)

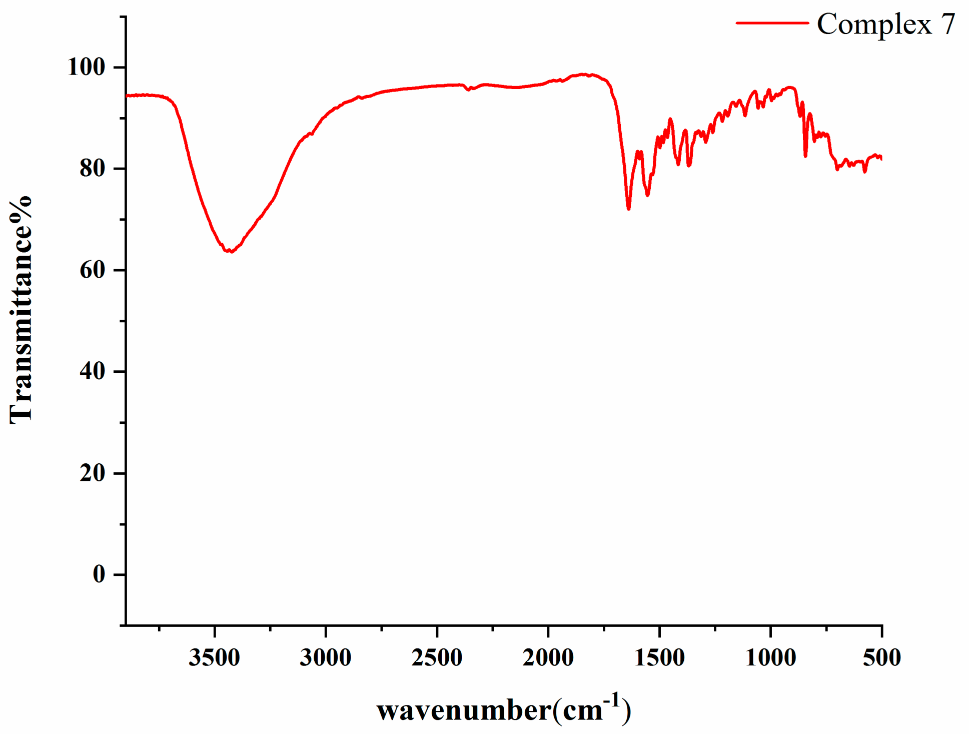

Complex 7
Transmittance%
100
80
60
40
20
0
3500
3000
2500
2000
1500
1000
500
wavenumber(cm-1)

(g)

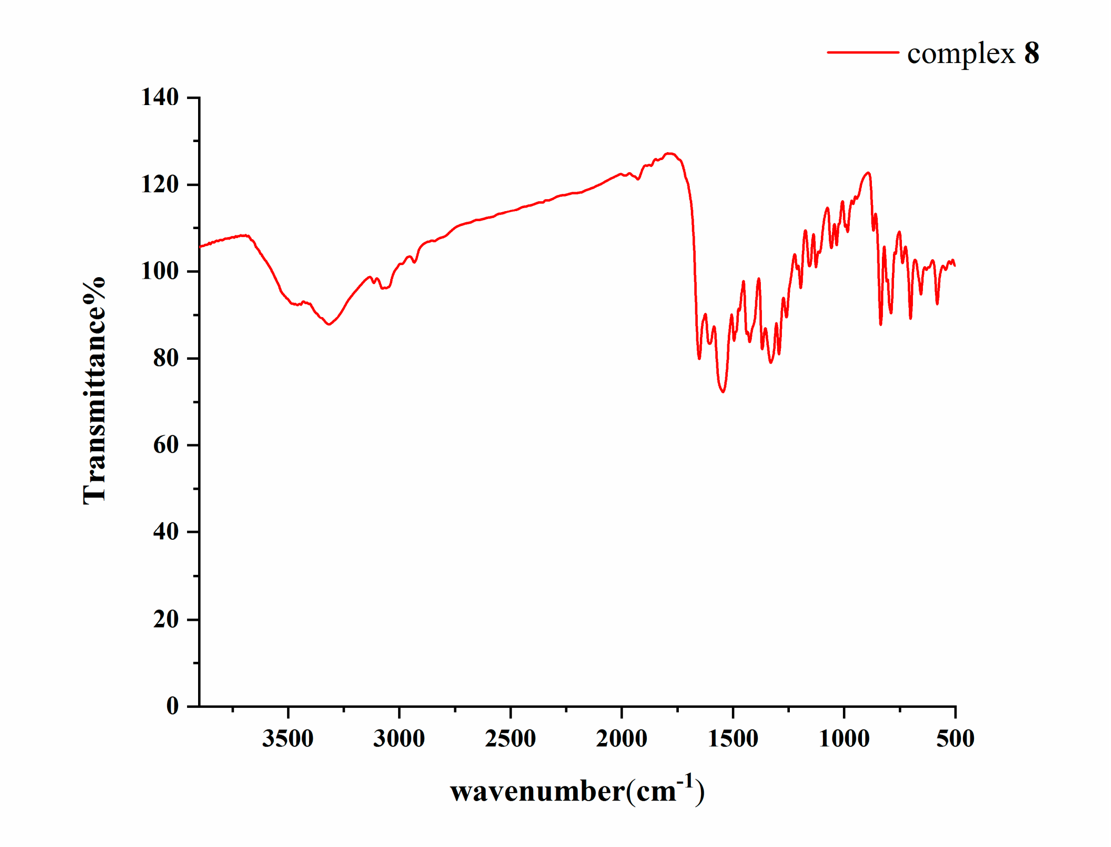

(h)

Figure S26. FTIR spectra of complex (a) **1**, (b) **2**, (c) **3**, (d) **4**, (e) **5**, (f) **6**, (g) **7** and (h) **8**.

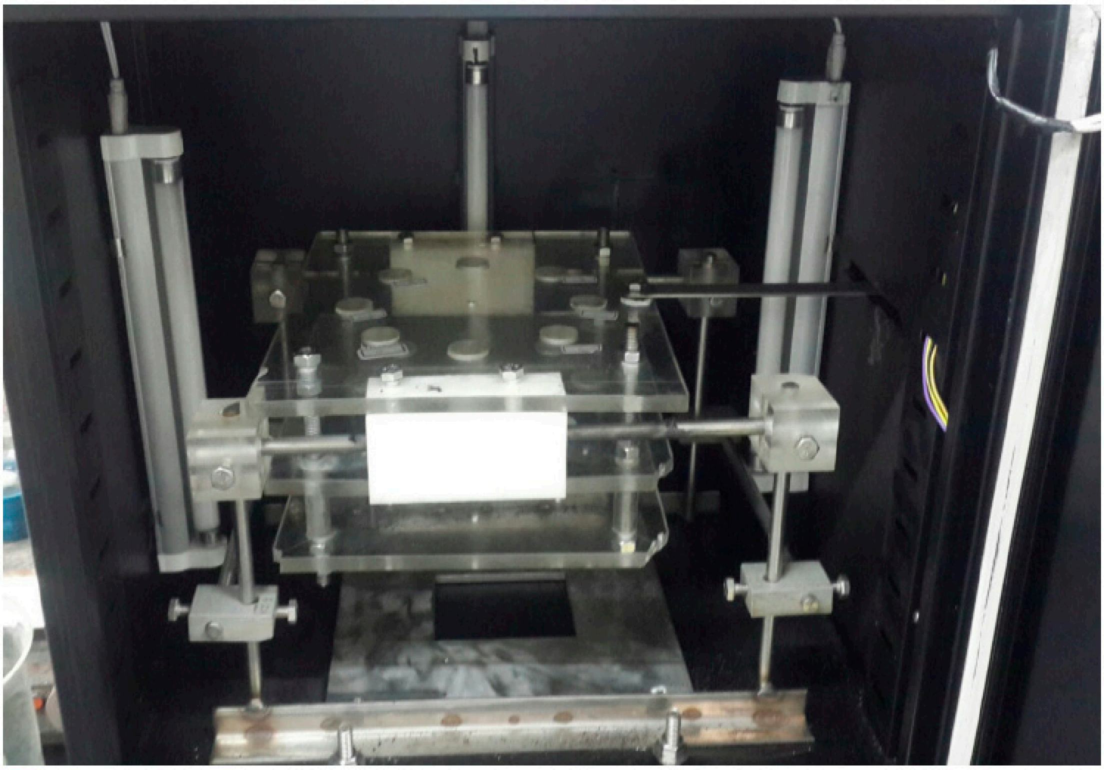

Figure S27. A photograph showing the internal side of a black photodegradation box.
